# Do I need to know my patient’s sexual orientation and gender identity? Insights from Swiss primary care physicians

**DOI:** 10.1186/s12875-025-03126-z

**Published:** 2025-12-10

**Authors:** Tom Schädler, Patricia Lampart, David Schiltknecht, Alexander Ort, Rebecca Tomaschek

**Affiliations:** 1 School of Health Sciences, Institute of Public Health, Zurich University of Applied Sciences, Winterthur, Switzerland; 2 Faculty of Health Sciences and Medicine, University of Lucerne, Lucerne, Switzerland; 3 Center for Primary and Community Care, Faculty of Health Sciences and Medicine, University of Lucerne, Lucerne, Switzerland

**Keywords:** Sexual orientation, Gender identity, SOGI, Primary care, Sexual and gender minority health

## Abstract

**Introduction:**

Primary care physicians (PCPs) play a pivotal role in the Swiss healthcare system, serving as the first point of contact for diverse populations, including sexual and gender minorities (SGM). The collection and use of sexual orientation and gender identity (SOGI) information is increasingly recognized in public health as a tool to address health inequities. This study explores PCPs’ perspectives, practices, and perceived relevance of collecting, documenting, and using SOGI information in Swiss primary care.

**Methods:**

Semi-structured interviews with 11 PCPs in German-speaking Switzerland were conducted. The data were analysed via a hybrid thematic analysis approach that combined inductive and deductive coding, guided by the minority stress model and a health equity perspective.

**Results:**

The PCPs generally acknowledged the relevance of SOGI information in specific clinical contexts, particularly mental and sexual health; however, they saw limited relevance in routine care. Documentation practices were inconsistent, often informal, and constrained by binary electronic systems. Open communication was described as a facilitator, although discomfort and fear of stigmatization limited proactive inquiry. SOGI is often framed in behavioural rather than identity terms. Most participants had received little to no formal training on SGM health and expressed ambivalence about the need for further education. Structural barriers, assumptions, and lack of visibility of SGM patients further contributed to the limited use of the SOGI in clinical decision-making.

**Conclusion:**

Despite the growing awareness of the SOGI as a social determinant of health, it is still rarely addressed in Swiss primary care. Addressing documentation barriers, promoting inclusive communication, and embedding SOGI-related information in medical education are critical steps toward equitable, patient-centred care.

**Supplementary Information:**

The online version contains supplementary material available at 10.1186/s12875-025-03126-z.

## Introduction

Primary care physicians (PCPs) are fundamental to the Swiss healthcare system. They typically serve as the first point of contact and provide a broad range of person-centred services, including health promotion, chronic disease management, and psychological and social support [1–3]. In 2022, 73% of the population aged 15 years and older visited a PCP at least once [4]. Only 9% of primary care consultations resulted in a referral to specialists, highlighting the central role of PCPs in initial and ongoing care [5]. As of 2016, 37% of all insured individuals were enrolled in the general practitioner model, which requires patients to access care through a PCP – a proportion that has shown consistent growth [6]. Patient satisfaction is high, with over 90% of patients reporting that they are satisfied or very satisfied with their PCPs’ services [6]. Thus, PCPs play a critical role in providing holistic and continuous care, including sexual and gender minority (SGM) populations.

Sexual orientation (SO) and gender identity (GI), hereafter referred to as SOGI, are increasingly recognized as social determinants of health, alongside factors such as age, education, and occupation [7]. For SGM individuals, SOGI-related factors significantly shape healthcare experiences and contribute to health disparities. Switzerland’s lesbian, gay, bi, trans and queer (LGBTQ) population faces elevated risks for adverse outcomes, such as higher rates of suicidal ideation and self-harming behaviour among queer youth [8, 9]. Additional disparities include poorer sexual health, restricted healthcare access, greater exposure to discrimination and violence, and increased substance use [10]. Stigmatization and fear of discrimination compound these issues, creating additional individual and systemic barriers to care [11, 12]. The international literature echoes these findings, underscoring the urgency of addressing these barriers [13, 14]. In response to persistent discrimination, specialized LGBTQ health centres known as Checkpoints have been established in major Swiss cities as part of the Aids Federation.

The structural and psychosocial barriers that sexual and gender minorities encounter in healthcare can be meaningfully conceptualized through the minority stress model [15, 16]. This framework posits that in addition to general life stressors, LGBTQ individuals face additional, chronic, and socially based stressors, such as anticipated discrimination, internalized stigma, or the need to conceal one’s identity, which negatively affect both mental and physical health. In clinical interactions, these stressors may manifest in patients’ reluctance to disclose their sexual orientation or gender identity or in heightened vigilance and anxiety when navigating care environments [17]. These individual responses are embedded within broader structural forces. Accordingly, the Intersectionality-Informed Model of LGBTQ Health Equity Promotion [18] highlights the interplay of systemic, institutional, and interpersonal factors – including provider attitudes, training gaps, and cis-heteronormative assumptions – that can marginalize patients across multiple dimensions of identity. Together, these frameworks underscore the need for inclusive care practices that not only acknowledge but also actively address the unique stressors faced by sexual and gender minority patients in everyday primary care.

While inclusive healthcare requires system-level change, as mentioned above, individual providers play a crucial role in mitigating or inadvertently reinforcing minority stress. Primary care physicians are expected to follow evidence-based guidelines, yet their personal beliefs, knowledge gaps, and clinical routines inevitably influence how they engage with patients’ sexual orientation and gender identity. Clinical decision-making is shaped not only by medical knowledge but also by professional norms, time constraints, and perceived responsibilities [19]. In regard to the SOGI, discomfort, lack of training, or fear of offending may lead providers to avoid the topic altogether, thereby reinforcing invisibility and missing opportunities for stigma-free and patient-centred care [20–22]. Even well-intentioned providers may rely on implicit assumptions or heteronormative communication patterns, which can undermine the provider–patient relationship, reduce trust, and negatively affect clinical outcomes [23–25].

Despite growing attention to SGM health equity internationally, little is known about how PCPs in Switzerland perceive and engage with SOGI-related aspects of patient care. Given the central role of PCPs in the Swiss healthcare system, understanding their attitudes, practices, and potential challenges is critical to informing more inclusive care models. This qualitative study explores the Swiss PCP’s perspectives on the collection, documentation, and clinical use of SOGI information. Specifically, it examines (1) physicians’ experiences with sexual and gender minority patients (2), their strategies and perceived barriers in assessing and recording SOGI information, and (3) their views on the relevance and clinical utility of SOGI information in everyday practice.

## Methods

### Study design

This qualitative study employed semi-structured explorative interviews to investigate how primary care physicians (PCPs) in Switzerland perceive, assess, and document information on patients’ sexual orientation and gender identity (SOGI). The interview guide was developed on the basis of the study aims and adapted from a prior qualitative study on queer healthcare in pharmacy settings [26]. A shortened version of the guide (see Table 1) was shared with participants in advance to facilitate preparation. The full guide (translated into English) is included in the appendix.

**Table 1:** Shortened interview guide with study aims

Topics		Study aim
Introduction and ice breaker	- Experience with SOGI patients - Anecdotes	(1) PCPs’ personal perspectives
Inquiry and personal attitude	- Asking patients about SOGI - Strategies for asking - Expectations from SOGI patients	(2) Assessing and documenting SOGI information
Documentation	- Documentation practices - Barriers and Facilitators	(2) Assessing and documenting SOGI information
Utilization in healthcare delivery / patient care	- Relevant medical situations - SOGI as health determinant	(3) Relevance and utility of SOGI information for patient care

### Ethical considerations

Within the consent process, participants were informed that participation was entirely voluntary, and that withdrawal was possible at any time without the need to provide justification. Verbal assurances were given that all data would be handled confidentially and that the reporting of results would ensure anonymity. Verbal consent was obtained prior to each interview and audio recording. Data were stored securely on a password-protected computer.

### Participant recruitment

The participants were recruited via purposive snowball sampling. Initial contacts were made through the Center for Family Medicine and Community Care at the University of Lucerne (*n* = 7). To broaden recruitment, 49 emails were sent to PCPs listed on the online platform OneDoc [27] with practices located in the cantons of Lucerne, Uri, Schwyz, Nidwalden, Obwalden, and Zug, yielding two additional interviews. Further participants (*n* = 4) were recruited via LGBTQ + health centres (Checkpoints) in Bern, Basel, Zurich, and Geneva. In total, 13 interviews were conducted.

### Data collection

A total of 13 PCPs participated in the study. The interviews were conducted in Swiss German between March and June 2024, primarily via Zoom (*n* = 11), with two interviews held in person (one at the University of Lucerne, one in a clinical setting). The length of the interviews ranged from 38 to 68 min (M = 54 min). All the interviews were carried out by the same researcher, a male health sciences master’s student at the University of Lucerne, at the time of the study. At the start of each interview, the participants received a brief explanation of the study objectives and the purpose of the audio recording. Verbal consent for recording was obtained in all cases. One pilot interview was conducted via the same procedure and was deemed suitable for inclusion in the analysis, as only minor adjustments were made to the interview guide. Field notes were taken during each session. No participants withdrew or requested follow-up interviews. In total, 11 interviews were included in the final analysis. One interview recording failed, and it was decided not to repeat the interview to avoid introducing bias. One interview was excluded from the analysis, as the participant was working primarily in the emergency department of a cantonal hospital.

### Data analysis

The audio recordings were transcribed using noScribe [28], an open-source AI-based transcription software designed for qualitative research. The software operates entirely locally on the researcher’s computer and employs large language models to generate initial transcripts. To protect participant confidentiality, demographic and identifying data were obtained separately before the start of the audio recording. All transcripts were reviewed, corrected, and verified manually by the corresponding researcher to ensure accuracy and completeness.

The verified transcripts were then imported into MAXQDA [29] and analysed through a hybrid thematic analysis combining inductive and deductive coding, following the approach described by Fereday & Muir-Cochrane [30]. Initial codes were derived from both emergent patterns in the data and preidentified domains from the interview guide. Through iterative coding cycles and regular team discussions, these codes were refined and clustered into overarching themes. Consensus on final themes was reached collaboratively within the research team to enhance rigor and reflexivity. Selected quotations were translated into English to illustrate key findings. Although transcripts were not returned to participants for member checking, the analysis followed the COREQ guidelines [31] to ensure transparency and methodological accountability.

### PCP characteristics

Among the eleven participants, five identified as female, five as male, and one as nonbinary. Six described their sexual orientation as heterosexual, one as homosexual, and with four participants, this information did not arise during the interviews. The participants’ average age was 46 years. Nine PCPs were based in urban settings, one in a suburban area, and one in a rural region. Four physicians were recruited through LGBTQ + health centres (Checkpoints), suggesting greater familiarity with SGM patient needs. All but one participant reported prior experience in caring for transgender patients, and several had acted as the primary care provider during gender-affirming medical transitions. Table 2 summarizes participant demographics and practice characteristics.

**Table 2:** Characteristics of the participating PCPs

		PCP (*n* = 11)
Age in years – mean (SD)		45.8 (9.2)
Female – *n*		5
Nonbinary – *n*		1
Sexual orientation – *n*	Homosexual	1
	Heterosexual	6
	NA	4
Medical focus – *n*	Specialist in General Internal Medicine (FMH)	8
	Specialist Haematology (FMH)	1
	Specialist Infectious diseases (FMH)	2
Practice location – *n*	Rural	1
	Suburban	1
	Urban	9
Recruited through Checkpoint (LGBTQ + Healthcare Centre) – *n*		4

## Results

### Creating space rather than asking directly: relational approaches to SOGI

Analysis of the interviews revealed that PCPs tend to emphasize openness and trust over direct inquiry when addressing SOGI. Most participants viewed their role as creating space for disclosure rather than systematically asking. They described this approach as part of person-centred care, encouraging openness without pressuring patients and allowing identity-related topics to emerge organically over time. Several PCPs described encouraging openness while acknowledging the importance of respecting patients’ boundaries. As one PCP explained,

*“In general*,* I always think it’s good when people can be as open as possible with each other. That would be my appeal*,* so to speak*,* to minorities and their families: that they dare to speak up. However*,* in the end*,* everyone has the right not to say anything. In addition*,* the right to be reserved. If the trust isn’t strong enough yet*,* then it’s okay not to express it.” (PCP02)*.

While SOGI-related conversations were described as sensitive, they were not considered unusual in primary care settings. The interviewees described diverse strategies for initiating SOGI-related conversations, which are often influenced by personal preferences and professional experience. Four PCPs reported routinely asking indirectly about the SOGI during initial contact.

*“What you do during the initial consultation is that you ask for the full personal history*,* the medical background*,* and the social history. In addition*,* that simply includes things such as occupation*,* hobbies*,* and the relationship status. I consciously try to ask that openly*,* whether someone is in a relationship*,* and then whether they have children.” (PCP 08)*.

Others preferred to frame such questions with a brief explanation of their medical relevance, aiming to normalize the inquiry and minimize patient discomfort.

*“For example*,* in the context of preventive care*,* I usually ask that with an introductory sentence. ‘You see*,* for us*,* sexuality is also part of life*,* and that’s why I need to ask you a couple of things. Is that okay with you?’ I introduce with a sentence like that.” (PCP 01)*.

Across all the interviews, the PCPs emphasized the value of building trust over time. Several described long-term relationships as a key strength of primary care, facilitating more open and in-depth conversations about identity-related aspects of health.

*“That’s the strength of primary care: over time*,* people start to feel comfortable and can share more and more. In addition*,* when they do*,* it allows us to care better for them.” (PCP 07)*.

### Navigating uncertainty and linguistic sensitivity

Participants frequently reflected on moments of uncertainty when SOGI topics arose, particularly around language use and perceived etiquette. While many felt comfortable discussing diverse identities, they also described a persistent tension between wanting to show respect and fearing inadvertent mistakes. These reflections underscored how communication norms, generational differences, and social taboos shape comfort levels in everyday practice. One physician shared:

*“This is already about small talk. And somehow*,* it shakes you inside how quickly you can become unsure. I genuinely want to show people respect and handle it as well as possible. However*,* it always feels like you’re afraid of breaking some kind of etiquette.” (PCP10)*.

Several participants emphasized the broader social context of language use and identity. They expressed concerns about rising expectations to “say the right thing” and noted the lack of clear, accessible guidance, particularly regarding nonbinary patients.

*“Uncertain – or let me put it this way: it’s an area where you have to choose your words even more carefully. In addition*,* that’s simply because these are exceptional situations when I’m confronted with this.” (PCP10)*.

The PCPs highlighted that societal taboos surrounding sexuality continue to pose a barrier to openly addressing the SOGI in clinical settings. Several participants noted that generational or cultural factors can influence how such questions are received, which particularly affects older generations. Others pointed to discomfort among younger heterosexual cisgender men, who may react defensively when they are asked open-ended questions about their sexual partners, especially when the question is phrased in a gender-neutral way.

*“I think it’s still a taboo topic because it’s such a very*,* very small minority group. So actually*,* it would be right to ask. Absolutely. However*,* I believe that most people would still feel offended if I were to ask that. And especially with older patients*,* that’s a complete no-go*,* isn’t it? I’m quite certain that people over 60 would be very unsettled. They would find it quite strange if their primary care physician asked them something like that*,* I think*,* in most cases. So it’s a bit about reading between the lines – knowing or sensing which patient I can ask what*,* when*,* and how.” (PCP 08)*.

*“If you ask young cis men openly about their partners*,* some of them will respond with something like*,* ‘No*,* no*,* only women*,* of course.’ They sometimes feel uncomfortable when you implicitly assume that they have different-gendered sexual partners.” (PCP02)*.

Several PCPs reflected on how their own identities shaped interactions with patients, particularly in relation to SOGI topics. One senior physician remarked that age and appearance, such as gray hair, may confer a sense of legitimacy or trust, making it easier to address sensitive issues in consultations:

*“It also has to do with gray hair. With gray hair*,* it’s more acceptable to ask.” (PCP 01)*.

Additionally, another participant emphasized how belonging to the LGBTQ + community fostered increased awareness, comfort, and fluency when discussing sexual and gender diversity:

*“For me*,* this is an important topic mainly because it affects me personally. I’m lesbian*,* I’m in relationships with women*,* and that’s why I think I’m more sensitized to the topic and more familiar with the terminology.” (PCP 08)*.

Together, these reflections underscore the diverse ways in which provider characteristics – whether rooted in professional seniority or shared lived experience – can facilitate or sometimes complicate inclusive patient care.

### Between memory aid and medical record: how PCPs document SOGI

Accounts of documentation practices highlighted a pragmatic but inconsistent handling of SOGI information. Most physicians recorded relevant details informally within the social history, citing both technical and ethical constraints. Limited electronic record options and concerns about confidentiality meant that inclusive documentation often relied on personal judgment and improvised solutions. SOGI documentation typically forms part of broader contextual information about patients’ lives:

*“I document it at least as an entry in the medical record*,* as part of the social history. It’s not something that needs to be marked as ‘important’ or flagged to always be visible. I would definitely record it once. However*,* it’s on the same level as noting whether a patient lives with their family*,* lives alone*,* or works as an electrician.” (PCP 09)*.

Several participants noted that patients’ sexual orientation is often inferred through relational status, for instance, via the gender of a partner. In cases where no relationship is disclosed, providers often consider sexual orientation irrelevant.

Four PCPs reported documenting discrepancies between patients’ sex assigned at birth and gender identity, often using formal diagnosis codes. However, most encountered limitations within the electronic health records system, which allows only binary entries. For nonbinary patients or those with specific names or pronoun preferences, workarounds such as alerts or pop-up notes were common but were viewed as unsatisfactory:

*“We also work with an electronic patient information system*,* and it’s like – this may sound harsh – but ultimately*,* it’s designed as a warning field if there’s something special*,* like a patient having allergies or anything else. It pops up whenever you open the record. For example*,* I would write in there*,* ‘Please address as female*,* even if (sex) still officially says male.‘” (PCP08)*.

Eight PCPs emphasized that documentation primarily serves as a memory aid, which is particularly valuable in maintaining continuity when patients are seen irregularly. Knowing a patient’s relationship or identity context was perceived as a way to foster more personalized and trusting care:

*“If you write it down*,* the advantage is the same for everyone. You are simply prepared the next time. Whether I ask about the wife Silvia or the homosexual partner Matthias*,* the patient always finds it helpful when I know what their situation is.” (PCP04)*.

One challenge in documenting SOGI data was PCPs’ concern that sensitive information, once recorded, could be viewed by other providers and misinterpreted or lead to stigma. Several participants mentioned honouring these wishes, e.g., if a patient requests that certain information not be recorded:

*“And yes*,* you cannot avoid that 100% in the sense that if someone goes to another doctor*,* a specialist*,* or a hospital and that is now on the list of diagnoses*,* then someone there might still react inappropriately.” (PCP03)*.

### When relevance is “medically relevant”: situational engagement with SOGI

The interviews showed that physicians often engage with SOGI only when it appears directly linked to the clinical concern, most commonly in sexual or mental health contexts. This selective focus reflects a biomedical framing that narrows the perceived relevance of SOGI, potentially overlooking its importance for trust, communication, and holistic care. For example, asking about sexual orientation during treatment for a sprained ankle was deemed inappropriate and unnecessary:

*“It would be a bit strange if I were to ask them about their sexual orientation when they come in with a sprained ankle. Because at that moment*,* it is completely irrelevant to me.” (PCP05)*.

However, SOGI information becomes more pertinent when addressing sexual health, particularly in the context of STI prevention, screening, or preexposure prophylaxis (PrEP) use. In such cases, SOGI is often inferred through discussions of sexual behaviour, relationships, or risk contexts rather than being asked about directly:

*“I only ask directly about sexual orientation when it becomes medically relevant. This is the case mainly for sexually transmitted infections. Therefore*,* if someone comes in with symptoms*,* I just need to know whether the sexual contacts were with men or women because I need to look for different things.” (PCP08)*.

*“I mean*,* it’s probably a bit mean*,* but if I feel it’s obvious*,* I don’t ask. You know*,* someone who’s been on the PrEP with us for 10 years*,* to me*,* it’s clear – is a gay man.” (PCP03)*.

Perspectives on the relevance of the SOGI in clinical risk assessments have varied. Some PCPs associated non-heterosexual orientation and trans identity with increased STI risk due to perceived behavioural patterns:

*“Homosexual people and transgender individuals typically have more pronounced sexual risk behaviours*,* which also leads to a higher incidence of infectious diseases and sexually transmitted infections.” (PCP 11)*.

Others emphasized that risk is shaped more by individual practices than by identity labels:

*“I think that it is no longer the case that homosexuals are exposed to a greater health risk. I think that is something individual. You either do it safely or you do not. That is a personal attitude.” (PCP02)*.

A few PCPs also noted the relevance of SOGI in mental health care, referencing both general trends and specific patient experiences:

*“Of course*,* there are more psychiatric comorbidities and substance use among individuals from sexual minorities. “(PCP 07)*.

*“It truly becomes relevant when it leads to a health problem. When it has psychological effects*,* individuals may feel mentally burdened by their gender identity or relationships. Then*,* it becomes a medically relevant issue for me.” (PCP 02)*.

One participant speculated that SOGI topics are underrepresented in general primary care because affected individuals may seek care elsewhere. One PCP stressed the continued importance of queer-specific services such as Checkpoints, which offer lower-threshold, identity-affirming environments:

*“Yes*,* definitely. As the study also mentioned*,* mental health is significantly worse. As long as medicine is not queer-sensitive and queer-inclusive or trans-sensitive and trans-inclusive*,* places such as a checkpoint are simply needed. It’s low threshold; they come there. Because otherwise*,* they go to the doctor less often*,* diagnoses are more likely to be missed*,* and they’re already struggling with their mental health.” (PCP 03)*.

Referrals to such centres were mentioned, particularly in complex psychosocial or *trans*-related cases:

*“And I also refer people with specific mental health concerns or with transgender-related questions to Checkpoint Zurich*,* where there are specialized psychologists who deal specifically with these issues.” (PCP 05)*.

More specialized PCPs, including those with experience in managing hormone therapy or gender-affirming procedures, described how trans patients’ sex assigned at birth remains clinically relevant. For example, in cardiovascular risk assessments or determining screening needs:

*“She naturally has a different heart risk because she was born male. You need to know whether she still has a prostate or a uterus. Well*,* she doesn’t have a uterus*,* but whether she still has the prostate*,* and how all that affects things. Prostate cancer can still develop. Then*,* there’s the whole issue with hormones. What is the effect on cholesterol and blood pressure? That’s definitely something that needs to be considered for somatic health.” (PCP 07)*.

*“Exactly*,* these men*,* for example*,* I have to refer for a gynaecological exam*,* or at least I need to make sure that the preventive examination is being done.” (PCP 06)*.

### Learning by doing: evolving awareness and informal education

Several participants highlighted a lack of formal education on SOGI topics during medical training. Only few expressed interest in SGM-focused modules, many preferred the integration of such content into existing continuing education formats, as conferences or interprofessional trainings. A more experienced participant explained how they are informing other colleagues:

*“No*,* the training courses are all internal to Checkpoint. I feel more like we are the ones who provide such training courses.” (PCP 03)*.

Reflections on knowledge and training revealed a pattern of informal, experience-based learning. Many participants described developing greater awareness through patient encounters, peer discussions, and personal reflection rather than through formal education. Several emphasized that structured training and interprofessional exchange could strengthen inclusive practice and normalize SOGI topics within primary care. One participant shared how SGM-related topics still sparked surprise of confusion among colleagues:

*“In my quality circle*,* the other GPs asked*,* is there such a thing now? ‘Ah*,* it is called nonbinary. I read about it in the Tages-Anzeiger.’ ‘Yes*,* my daughter also has someone like that at school.’ ” (PCP03)*.

In addition to formal training, many PCPs described learning through self-directed education, experience, and reflection. Six participants discussed how their attitudes and understanding had evolved over time. One recounted a key insight that reshaped their perspective on gender identity:

*“I still remember that 15 years ago*,* I wondered when I know if a person has a different gender identity than the sex they were assigned. Until I finally understood*,* aha*,* when the person tells me that*,* that is how it is*,* but it took me forever to realize that. You cannot measure it*,* you do not need a psychiatrist*,* you do not need an endocrinologist*,* you do not need anything*,* you just need a person to tell me that is the way it is.” (PCP06)*.

## Discussion

### Summary of the main findings

This study explored how Swiss primary care physicians (PCPs) perceive, document, and use sexual orientation and gender identity (SOGI) information in clinical care. While some providers acknowledge the relevance of the SOGI in mental and sexual health, most consider it secondary or even irrelevant for routine care. Documentation was inconsistent, typically informal, and limited by binary electronic health record systems. Although open communication was valued, several PCPs reported discomfort when SOGI emerged during consultations. Training on SOGI-related topics was minimal, and there was limited demand for further education.

These findings point to missed opportunities for inclusive care and are best understood through the lens of the Minority Stress Model, which conceptualizes how stigma and structural invisibility contribute to the probability of poorer health outcomes among sexual and gender minority (SGM) individuals [15, 16]), and an intersectional health equity lens, which highlights how overlapping social identities (e.g., sexual orientation, gender identity, age, migration background) shape access to care and provider interactions [18].

### The limited integration of SOGI in Swiss primary care

SOGI can be a vital component of patient identity and a relevant social determinant of health. When included as part of a holistic primary care approach, SOGI data inform tailored prevention strategies, improve communication, and most importantly, create an inclusive and welcoming environment that fosters trust. As discussed by Dichter and Ogden [32], the patient’s wishes and privacy must always be prioritized, and SOGI data should be shared or used only when relevant. This emphasis on respectful boundaries aligns with how many PCPs in our study reported using introductory sentences to normalize such inquiries and reduce potential discomfort.

Nevertheless, several PCPs reported never having knowingly treated an SGM patient. This raises the question of whether this reflects the actual absence of such patients or rather stems from provider assumptions, limited awareness, or patients’ reluctance to disclose their identity. Notably, some physicians equated sexual orientation to nonmedical characteristics such as hair color, framing it as irrelevant in a clinical context. Others inferred sexual orientation on the basis of indirect cues such as medication use, e.g., interpreting PrEP prescriptions as indicators of homosexuality, demonstrating the role of implicit assumptions in the absence of open discussion. These patterns echo findings from oncology, where discomfort and a limited understanding of the relevance of SOGI were cited as major barriers to documentation and engagement [32, 33].

In primary care, where long-term provider‒patient relationships with patients are common, a holistic view is particularly important. Maragh-Bass [34] emphasizes that SOGI data form a critical part of a broader social history, alongside factors such as race, disability status, or chronic illness. These elements should be interpreted together to understand the patient’s lived experience. In the Swiss context, the importance of this perspective is underscored by public preferences: 65% of the population reports that they would turn to their PCP first in the case of a health concern. However, this figure decreases to 50% among 18–34-year-olds, with 23% in this age group indicating that they would prefer to use a health app instead. Notably, trust in PCPs remains stable across urban and rural regions [35]. These patterns suggest that while PCPs retain a key role, particularly among older patients, the relevance of inclusive and identity-aware care is growing, especially in light of generational shifts.

The need for a more holistic approach also becomes apparent in cases where SOGI is overlooked simply because patients are not currently in a relationship or when sexuality is not spontaneously brought up. In such cases, missed opportunities for understanding the broader psychosocial context can affect the quality of care and reinforce SGM invisibility.

### When inclusion depends on context: conditional engagement with SOGI

The PCPs in our study described SOGI as relevant in the context of mental health. This is consistent with the broader role that PCPs play in providing first-line treatment for psychiatric conditions, including the prescription of psychotropic medication. In the U.S., approximately 30% of patients treated in primary care settings receive mental health support [36], and Switzerland has implemented measures to promote early detection and collaborative care for psychological disorders [37]. Considering the disproportionate mental health burden among SGM populations [10, 24], awareness of SOGI-related stressors is essential for appropriate care. The minority stress framework highlights how chronic exposure to stigma, concealment, and social exclusion can manifest in mental health symptoms. When PCPs are unaware of these dimensions, they may underrecognize root causes or fail to provide appropriate referrals or interventions.

SOGI is equally relevant in sexual health contexts, including STI prevention, contraception, PrEP access, and sexual functioning. Several PCPs indicated that they only inquired about sexual orientation or gender identity when discussing specific risks or symptoms, often framing such questions in behavioural rather than identity terms. While this can be effective in tailoring STI screenings, it may also limit the scope of discussion and reinforce the idea that SOGI matters only in narrowly defined clinical moments. Importantly, some participants associated certain SOGI identities, particularly homosexuality or trans identity, with higher-risk behaviour. Although clinical vigilance is important, equating identity with risk may perpetuate stigma if not grounded in individualized assessments. Others emphasized that risk behaviours vary between individuals and are not determined by identity labels alone. This more nuanced perspective aligns with health equity principles and highlights the importance of asking open-ended, behaviour-based questions without making assumptions. The findings also suggest that younger heterosexual cisgender men may react defensively to gender-neutral inquiries about partners, further underscoring the need for communication strategies that reduce discomfort and promote trust. Inclusive sexual health conversations not only support prevention but also serve to validate patients’ identities and normalize diversity in clinical settings.

Despite its relevance, SOGI remains under-addressed in daily practice. One participant suggested that SGM patients may avoid general primary care settings and instead seek support from queer-specific services such as Checkpoints, which provide low-threshold, identity-affirming care. In such cases, PCPs may be unaware of the presence or needs of SGM patients in their own practices, perpetuating the cycle of invisibility. Moreover, more experienced or specialized PCPs described cases where transgender patients’ sex assigned at birth was clinically relevant for treatment decisions such as cardiovascular risk management or cancer screening. These examples underscore the medical importance of context-specific SOGI knowledge beyond assumptions or visual cues.

Ongoing initiatives, – such as those supported by the Swiss National Science Foundation – aim to strengthen the integration of sex and gender aspects into health research, public health, and clinical education. Parallel efforts by interuniversity working groups promote the inclusion of sex and gender topics in Swiss medical curricula [38, 39]. Evidence from other settings suggests that exposure to LGBT-specific health content during medical training improves both attitudes and clinical practices [40–42]. Taken together, these findings indicate that knowledge gaps regarding SOGI could be addressed through targeted, system-level strategies without requiring major structural overhauls in medical education.

### Informal and constrained documentation practices

Holistic and inclusive care includes respectful documentation of SOGI information. Most PCPs reported entering SOGI-related details in their social history, typically during initial consultations. While this documentation serves primarily as a memory aid, its informal and inconsistent nature means that important information may be overlooked during follow-ups or in shared-care settings. Transgender identity was sometimes recorded in the diagnosis list, particularly when it was clinically relevant. However, this practice raises tensions between clinical pragmatism and concerns about stigmatization. The recent reclassification of gender incongruence in the ICD-11 as a sexual health issue rather than a mental disorder represents an important shift [43]. Nonetheless, challenges remain in applying this reclassification in everyday practice without unintentionally reinforcing pathologization.

In addition to technical systems, structural barriers also shape documentation practices. Several participants described binary-only electronic systems as incompatible with gender-diverse realities. Workarounds such as pop-up alerts were used to flag names or pronouns, but these solutions were often unsatisfactory. The participants also expressed concern about inadvertently violating patient confidentiality, particularly when documentation is visible to other healthcare providers who may lack training or sensitivity. Some patients reported that SOGI information was not recorded, reflecting their awareness of the potential for discrimination.

LeClair et al. [44] identified further barriers to SOGI data collection, including communication breakdowns with interpreters and discomfort during cross-generational interactions. Although the interpreter issued did not arise in our interviews, language emerged as a clear challenge, particularly in Swiss German, where no established pronouns for nonbinary individuals exist. The PCPs described small talk and routine conversations as potential sources of anxiety, with some expressing uncertainty about how to speak inclusively without overstepping. Interestingly, generational gaps were sometimes perceived as advantages. In contrast to the findings of LeClair et al. [44], several participants noted that an age difference could reduce the risk of misinterpretation, as patients were less likely to perceive neutral questions as flirtatious or intrusive.

### Good intentions under structural limits: the role of provider assumptions

Both implicit and explicit provider attitudes shape clinical practice and can significantly affect the quality of care for SGM patients. As Hamed et al. [45] note, racism in healthcare manifests through stereotyping, unequal treatment, and dismissive communication, patterns that also apply to sexual and gender minority populations. In our study, several PCPs expressed support for respectful care while simultaneously reporting avoidance behaviours, knowledge gaps, or reliance on assumptions. These findings align with prior research showing that even well-meaning providers may unintentionally reproduce heteronormative or cis-normative assumptions [24, 25]. The perception that SOGI is only clinically relevant when directly tied to the presenting concern was widespread. However, this approach risks the absence of unexpressed needs, especially given the tendency among SGM patients to conceal identities due to anticipated stigma. Brooks et al. [23]. have shown that nondisclosure is often linked to prior negative experiences or fears of being judged. Without proactive efforts to create safe disclosure environments, these patterns are unlikely to change.

A Swiss study revealed that male patients are generally open to discussing their sexual health but prefer when PCPs take the lead [46]. This suggests that provider hesitation may limit opportunities for meaningful dialogue and intervention. Indeed, the participants in our study reported that sexuality and SOGI were addressed only when “relevant”, a framing that reinforces invisibility unless it was linked to disease or risk. These practices stand in tension with growing public acceptance of sexual diversity [47] and the evolving expectations of younger patient populations.

### Strengths, limitations, and future directions

This study offers in-depth insights into how Swiss PCPs perceive, engage with, and document SOGI information. A key strength lies in the semi-structured qualitative design, which allows for the open-ended exploration of sensitive topics and enables nuanced accounts of clinical reasoning, professional beliefs, and contextual influences. The sample included both rural and urban PCPs, as well as varying levels of exposure to SGM patients, enhancing the breadth of the perspectives captured.

However, several limitations must be acknowledged. First, the sample size was small and purposive, which limits generalizability. Recruitment proved difficult, suggesting potential selection bias: participants may have been more open, interested, or comfortable discussing SOGI than the broader PCP population. This is underscored by the explicit refusal of one invited PCP who expressed strong anti-SGM views. Second, social desirability bias cannot be ruled out. Given the increasing attention to diversity and inclusion in medical contexts, participants may have offered responses they perceived as socially appropriate.

From a methodological perspective, while thematic saturation was likely approached, we cannot definitively claim that it was achieved. As qualitative research is context-bound and interpretive, future studies should aim for more diverse sampling, including non-responders or those uncomfortable with SGM topics. Additionally, we acknowledge our own positionality as researchers committed to health equity and inclusive care. While this informed our choice of frameworks (e.g., intersectionality, minority stress), it may also shape our interpretation of findings.

Future research should build on these findings by exploring the perspectives of SGM patients regarding their experiences in Swiss primary care, particularly with respect to disclosure, safety, and trust. Longitudinal or intervention-based studies could evaluate the impact of SOGI-specific training programs on provider confidence and patient outcomes. Moreover, studies should investigate systemic factors such as electronic medical record structures, billing codes, and organizational policies that may enable or hinder inclusive documentation. Finally, future work could examine how intersections of SOGI with other dimensions, such as race/ethnicity, migration background, disability, or age, affect visibility, recognition, and treatment in primary care, further operationalizing intersectionality in clinical research and practice.

### Implications: toward inclusive and equity-oriented primary care

These findings point to an urgent need to embed SOGI awareness into routine primary care. This is not an optional add-on but rather a core component of inclusive, evidence-based practice. While many PCPs aim to offer respectful and patient-centred care, the inconsistent handling and documentation of SOGI information can unintentionally perpetuate disparities. This is exacerbated by providers’ discomfort, system limitations, and a lack of formal training.

Clear clinical guidance is needed to support PCPs in assessing and documenting SOGI information. Starting with how to ask, when to ask, and how to respond to disclosures. Electronic documentation systems must be updated to include nonbinary and flexible gender options. These technical improvements should be combined with educational reforms. Expanding medical curricula to include SGM-specific health issues, such as mental health risks, sexual health disparities, and minority stress, can improve both competence and confidence in delivering inclusive care.

Importantly, these efforts should not be limited to physicians. Inclusive care must be supported by all members of the primary care team, including nurses, medical assistants, and administrative staff. Interprofessional training, awareness campaigns, and local champions can help promote a culture of inclusion at every level of care delivery. Recent Swiss initiatives funded by the National Science Foundation, as well as interuniversity curriculum reform projects, represent key opportunities to support this transformation.

## Conclusions

This study provides insights into how the Swiss PCPS perceive, engage with, and document SOGI information in clinical practice. While the participants expressed a general commitment to patient-centred care, SOGI was often seen as relevant only in specific contexts, such as mental or sexual health. Routine inquiry and documentation remain inconsistent and shape by assumptions, discomfort, and system-level limitations. These practices risk reinforcing invisibility and health disparities for SGM populations. In efforts to promote equity in healthcare, our findings underscore the importance of normalizing inclusive communication, improving structural conditions for documentation, and embedding SOGI-related content in medical training. To support a truly holistic approach to care, PCPs must be equipped with the tools, knowledge, and institutional backing needed to engage respectfully and competently with diverse patient identities.

## Supplementary Information

Supplementary Material 1.

## Data Availability

The datasets used and/or analysed during the current study are available from the corresponding author upon reasonable request.

## Abbreviations

GI: Gender identity
LGBTQ: Lesbian, gay, bi, trans, queer
PCP: Primary care physician
PrEP: Preexposure prophylaxis
SGM: Sexual and gender minorities
SO: Sexual orientation
SOGI: Sexual orientation and gender identity
