# Supplementary material for: Do I need to know my patient’s sexual orientation and gender identity? Insights from Swiss primary care physicians
Source: BMC Prim Care. 2025 Dec 10;27:11. doi: 10.1186/s12875-025-03126-z (PMC12801783; doi:10.1186/s12875-025-03126-z)
Supplement: Supplementary file 1 — Supplementary Material 1. [file 12875_2025_3126_MOESM1_ESM.docx]

**Interview Leitfaden (DE)**

| **Thema** | **Leitfrage** |
| --- | --- |
| **ICE Breaker & Einführung** | Haben Sie Erfahrungen mit Patient:innen, die einer Minderheit innerhalb der sexuellen Orientierung oder Geschlechtsidentität angehören?  **Folgefrage(n)**  Können Sie mir von diesen Erfahrungen berichten; waren sie positiv oder negativ?  Gibt es Unterschiede zu Patient:innen, die keiner Minderheit innerhalb der SO oder GI angehören?  Wie oft sehen Sie Patient:innen, die einer Minderheit angehören? |
| **Erfragung**  **& persönliche Einstellung** | Erfragen Sie die sexuelle Orientierung oder Geschlechtsidentität Ihrer Patienten?  **Folgefrage(n)**  Gibt es spezielle Szenarien, in denen Sie direkt nach der SO oder GI fragen?  Wie fragen Sie ihre Patient:innen nach ihrer SO oder GI?  Gibt es Herausforderungen, wenn Sie Ihre Patient:innen nach ihrer SO oder GI fragen?  Fühlen Sie sich wohl, wenn sie nach der SO oder GI fragen?  Gibt es Strategien oder Hilfsmittel, um eine angenehme Umgebung zu schaffen?  Erwarten Sie von Ihren Patienten, dass Sie sie über die SO oder GI informieren, wenn sie einer Minderheit innerhalb der sexuellen Orientierung oder Geschlechtsidentität angehören? |
| **Dokumentation** | Dokumentieren Sie die sexuelle Orientierung und Geschlechtsidentität Ihrer Patient:innen?  **Folgefrage(n)**  Wenn ja: Wie? Was sind Hindernisse für die Dokumentation? Was sind Förderfaktoren für die Dokumentation?  Wenn nein: Wieso nicht? Was könnte Sie dabei unterstützen diese Information zu dokumentieren?  Sehen Sie einen Mehrwert darin, diese Information zu dokumentieren?  Sehen Sie Gefahren darin, diese Informationen zu dokumentieren? |
| **Auswirkungen auf die Versorgung** | Können Sie sich Situationen vorstellen, in denen das Wissen über die sexuelle Orientierung oder Geschlechtsidentität Ihrer Patient:innen, Auswirkungen auf die Behandlung hat?  **Folgefrage(n)**  In welchen Bereichen der medizinischen Versorgung sehen Sie einen Mehrwert dieses Wissens?  Glauben Sie, dass die SO oder GI die Gesundheit Ihrer Patient:innen beeinflusst?  Welche Leistungen bieten Sie für Patient:innen die einer Minderheit innerhalb der SO oder GI angehören an?  An welche weiterführenden Dienste verweisen Sie?  Wie können Hausärzte Ihrer Meinung nach dazu beitragen, die Gesundheitsversorgung von Patient:innen, die einer Minderheit innerhalb der SO oder GI angehören, zu verbessern? |
| **Ausbildung & Training** | Waren Themen zur sexuellen Orientierung oder Geschlechtsidentität Thema im Medizinstudium?  Werden diese Themen im Rahmen von Fortbildungen behandelt?  **Folgefrage(n)**  Sind diese Fortbildungen von Interesse für Sie?  Haben die Informationen Einfluss auf Ihren klinischen Alltag?  Fühlen Sie sich kundig in den Themen?  Gibt es Leitlinien oder Tools, die Sie zu Rate ziehen können?  Wie stellen Sie sich Tools oder Informationen zu diesem Thema vor?  Wo würden Sie nach weiteren Informationen suchen? |
| **Abschliessende Fragen** | Vielen Dank, dass Sie sich die Zeit genommen haben, um mit mir über die SO oder GI in der Grundversorgung zu sprechen. Gibt es sonst noch etwas, das ich wissen sollte? |

**Interview guide (EN)**

| **Theme** | **Key question** |
| --- | --- |
| **ICE Breaker & Introduction** | Do you have experience with patients who belong to a minority group in terms of sexual orientation or gender identity?  **Follow-up question(s)**  Can you tell me about these experiences; were they positive or negative?  Are there any differences compared to patients who do not belong to a minority group in terms of SO or GI?  How often do you see patients who belong to a minority group? |
| **Enquiry & personal attitude** | Do you ask your patients about their sexual orientation or gender identity?  **Follow-up question(s)**  Are there specific scenarios in which you ask directly about SO or GI?  How do you ask your patients about their SO or GI?  Are there any challenges when asking your patients about their SO or GI?  Do you feel comfortable asking about SO or GI?  Are there any strategies or tools you use to create a comfortable environment?  Do you expect your patients to inform you of their SO or GI if they belong to a minority within sexual orientation or gender identity? |
| **Documentation** | Do you document the sexual orientation and gender identity of your patients?  **Follow-up question(s)**  If yes: How? What are the obstacles to documentation? What are the factors that promote documentation?  If no: Why not? What could help you to document this information?  Do you see any added value in documenting this information?  Do you see any risks in documenting this information? |
| **Impact on care** | Can you think of situations in which knowledge about your patients' sexual orientation or gender identity has an impact on their treatment?  **Follow-up question(s)**  In which areas of medical care do you see added value in this knowledge?  Do you believe that SO or GI affects your patients' health?  What services do you offer to patients who belong to a minority within SO or GI?  What additional services do you refer them to?  In your opinion, how can family doctors help improve healthcare for patients who belong to a minority within SO or GI? |
| **Education & Training** | Were topics related to sexual orientation or gender identity covered during your medical studies?  Are these topics covered in continuing education courses?  **Follow-up question(s)**  Are these continuing education courses of interest to you?  Does the information influence your daily clinical practice?  Do you feel knowledgeable about these topics?  Are there any guidelines or tools you can refer to?  How do you imagine tools or information on this topic?  Where would you look for further information? |
| **Final questions** | Thank you for taking the time to talk to me about SO or GI in primary care. Is there anything else I should know? |
